# Suspected Posttraumatic Mandibular Coronoid Hyperplasia and Condylar Hypoplasia: Two Case Reports and Literature Review

**DOI:** 10.1155/crid/8940005

**Published:** 2025-08-26

**Authors:** Areeg Elmusrati, Mariela Padilla

**Affiliations:** Herman Ostrow School of Dentistry, University of Southern California, Los Angeles, California, USA

**Keywords:** condylar hypoplasia, coronoid hyperplasia, facial asymmetry, limited mouth opening

## Abstract

Changes in the size of the coronoid process, due to hyperplasia or hypoplasia, may interfere with the normal range of mouth opening. Coronoid hyperplasia is a rare oral and maxillofacial disease which might result in progressive limitation of mouth opening due to the impingement of an abnormal elongated mandibular coronoid process on the zygomatic arch. This condition presents with clinical challenges due to its nonspecific nature of complaints that present in patients. It is characterized by gradual painless restriction of mouth opening. Currently, the exact etiopathology still remains inconclusive; however, genetic, hormonal, inflammatory, or traumatic influences have been reported. In this paper, the authors present two case reports of adults with suspected unilateral posttraumatic coronoid hyperplasia and developmental condylar hypoplasia that have a history of physical trauma inflicted during adolescence. Provided is a succinct update of literature to highlight the etiopathogenesis, significance of accurate diagnosis, and tailored treatment modalities in achieving favorable outcomes to restore function and esthetics.

## 1. Introduction

The temporomandibular joints (TMJs) are bilateral, diarthrodial joints formed by the articulation of the movable mandible to the fixed temporal bone of the cranium [1]. The articulating condylar cartilage is an essential mandibular growth center, and any deviations or disruptions in growth of the condylar structure can affect both occlusal function and facial esthetics. Hypoplasia of the condylar process can be congenital or acquired. Congenital condyle hypoplasia can be uni- or bilateral and is usually associated with systemic disorders originating from the first and second branchial arches. These genetic conditions include Treacher Collins, Goldenhar, auriculocondylar, Morquio, and Proteus syndromes. Secondary or acquired condylar atrophy can also be caused by systemic factors as certain autoimmune diseases as rheumatoid arthritis and systemic sclerosis. In addition, unilateral condyle hypoplasia has been reported to be caused by a traumatic event during childhood or adolescence, TMJ dysfunction, local infections, and irradiation [2].

The mandibular coronoid process is an anatomical structure that serves as a point of attachment of the temporalis, buccinator, and anterior insertion of the masseter muscles. Apart from this, the coronoid has no major functional role in the jaw and can be surgically removed as a source of bone graft or due to pathology without causing any significant limitations [3].

Mandibular coronoid hyperplasia is a relatively uncommon congenital or developmental condition and one of the rare causes of progressive restriction of mouth opening. This abnormal macroscopic overgrowth leads to impingement of the enlarged or elongated coronoid process on the medial surface of the zygomatic arch, limiting mouth opening [4, 5]. Coronoid hyperplasia can present in one or both sides. The unilateral condition can involve facial asymmetry with deviation of the mandible to the affected side. In 1853, Langenbeck introduced coronoid hyperplasia, and Jacob was the first to report a case of limited mouth opening due to an increased coronoid process size in 1899 [6].

Several studies have reported that following microscopic analysis, the unusual elongation of the mandibular coronoid process was composed of histologically normal osseus tissue [7]. Progressive restriction of mouth opening is the most common and consistent symptom [4, 5]. However, various clinical features have been reported, including facial asymmetry presenting with swelling or protuberance of the zygomatic or preauricular region, which is an indication of the hypertrophic growth of the coronoid process. In addition, patients may complain of painless progressive restriction of their mouth opening or even as severe as trismus, due to the impingement of an enlarged coronoid to the zygoma. It has been reported that affected individuals complaining of limited mouth opening accompanied with pain or crepitation are evident in 7% and 8%, respectively [8]. In infants, restricted mouth opening may lead to several complications, such as compromised feeding and breathing difficulties [9]. The gradual onset and progression of symptoms further complicate reaching a definitive diagnosis, and without a thorough clinical and radiological evaluation, the condition may be confused with TMJ disorders [10, 11]. These conditions represent challenging dysfunctions, provoking diagnostic and therapeutic uncertainties for clinicians, emphasizing the need for a comprehensive understanding of the hyperplastic coronoid process etiology, clinical manifestations, diagnostic approach, and treatment strategies.

The etiopathology of this bony hyperplasia remains unclear, but numerous theories have been proposed [4, 5]. Coronoid process hyperplasia's developmental origins are multifactorial, and heterogenous etiological considerations have been suggested. While TMJ disorder, neoplasia, and trauma, especially to the zygomatic arch, are thought to be a contributing factor in some cases, a substantial percentage of cases reported are idiopathic in nature [4]. Hyperactivity of the temporalis muscles has been hypothesized to be a key source of coronoid lengthening. The continuous uncontrolled muscular contractions result in excessive mechanical stress on the coronoid process, stimulating osteogenesis.

Radiological analysis plays a key role in identifying coronoid hyperplasia as well as condylar hypoplasia. It provides necessary visualization to the morphology and extent of the bony defect. Conventional imaging such as orthopantomogram (panoramic radiography) and lateral cephalometric analysis may reveal the relation of the coronoid and condylar processes with proximal craniofacial structures. However, advanced techniques such as cone-beam computed tomography (CBCT) provide enhanced spatial resolution of the oral and maxillofacial structures. CBCT produces a precise three-dimensional reconstruction, aiding in a comprehensive preoperative evaluation [11, 12].

Presently, after the patient has stopped growing, skeletal deformities of the condyle can be corrected by jaw surgery with/out genioplasty or unilateral mandibular augmentation [2]. However, the gold standard for management of coronoid process hyperplasia is surgical intervention through an extra- or intraoral approach. Partial bone removal (coronoidotomy) or complete excisions (coronoidectomy) have been performed [7, 12]. Eliminating the mechanical obstruction and restoring mandible functionality provide a conclusive solution to restricted mouth opening and facial asymmetry. The decision to proceed with a surgical approach depends on a spectrum of factors ranging from patient symptoms, dimensions of coronoid overgrowth, presence of genetic factors, and possibility of recurrence [13]. These case reports are aimed at describing posttraumatic coronoid hyperplasia with condylar hypoplasia in a 39-year-old male and a 27-year-old female, emphasizing the clinical manifestations and diagnostic challenges, with particular reference to initial radiographic evaluation using orthopantomography. While reviewing the literature, the authors seek to highlight the importance of better understanding of this developmental disorder. Both patients signed a consent for deidentified publication of their cases, and the reports follow CARE guidelines. The examining clinician is calibrated for TMJ assessment by the USC Orofacial Pain Clinic protocols.

## 2. Case Report #1

A 39-year-old male of European origin was referred to the Herman Ostrow School of Dentistry Orofacial Pain Clinic to evaluate the TMJ for limited mouth opening and occasional clicking. During a recent visit to the dental office, the dentist found it challenging to proceed without using a bite block to extend his mouth opening during dental procedures. The patient's medical history was unremarkable (ASA Type 1); however, when he was 9 years old, he was hit by a skateboard on the left side of his face, which resulted in a nasal fracture, where he had to undergo reconstructive surgery. As a young adult, following this incident, he gradually noticed the development of bilateral clicking sounds of the TMJ and difficulty in opening his mouth wide.

**Figure 1:**
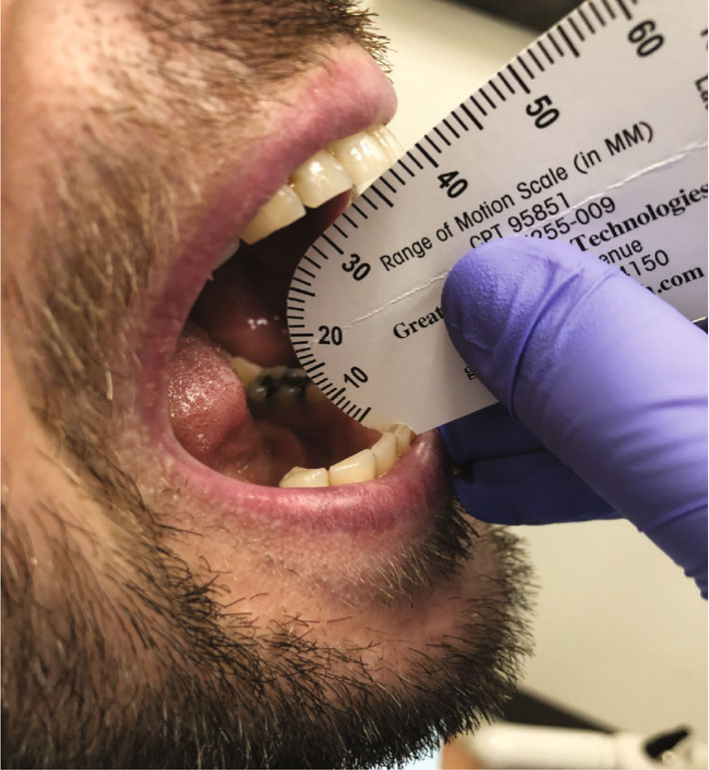
Extraoral photograph showing maximum mouth opening.

Extraoral examination revealed no noticeable swelling or facial asymmetry; however, no photographic analysis was conducted, limiting the ability to document and report on this variable. Masticatory muscles demonstrated no tenderness or pain upon digital palpation. However, mild pain was reported in the lateral capsule of the TMJ region, with a slightly higher pain and discomfort on the left side. Bilateral intracapsular sounds (clicking) were identified, and the mandible appeared to deviate to the left side on opening of the mouth. Importantly, there was limited range of mandibular movements; his maximal interincisal opening (MIO), both maximum assisted and unassisted, was 38 mm (Figure 1). His lateral movement of the mandible was also restricted to the left side being 6 mm and the right 8 mm. Intraoral normality of alveolar tissues was noted. The maxillary and mandibular dentition was well aligned, presenting with bilateral posterior tooth contact with no occlusal derangement.

**Figure 2:**
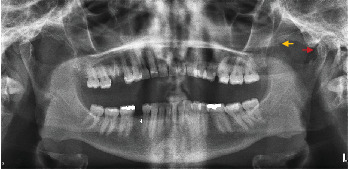
Orthopantomography of the patient. Left coronoid process hyperplastic (yellow arrow). Left condyle hypoplastic (red arrow).

**Figure 3:**
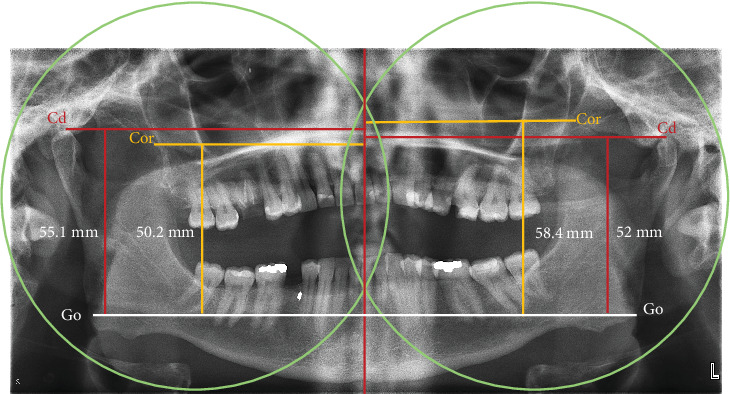
Levandoski method to assess coronoid hyperplasia. Vertical midline was drawn between the two intersections of both arcs (center of arcs of maxillary tuberosities/distal height of the second molars). Three horizontal lines were drawn perpendicular to the first line. 1—condyle (Cd) (red, Cd-midline) was determined from the tip of the condyle, 2—coronoid (Cor) (yellow, Cor-midline) was measured from the tip of the coronoid, and 3—gonion (Go, white) across the lower border of the symphysis of the mandible. The Go⁣′–Cor⁣′/Go⁣′–Cd⁣′ length ratio was then calculated. Right = 0.91 and left = 1.12.

An orthopantomogram was taken (Figure 2). Orthopantomograms provide a useful overview for initial assessment; however, a limitation of this imaging modality is the superimposition of the zygomatic arch, which can obscure the exact position of the upper portion of the coronoid process. Although the superimposing of the structures on the 2-D image exists, the image most probably revealed an enlargement of the left coronoid and an elongation of the tip of the coronoid process. Upon contralateral comparison, left condyle hypoplasia with mild flattening or bony erosion was also detected (Figure 2). There was no radiographic evidence of the development of any neoplastic growth in the coronoid process. The coronoid–condyle length ratio measurement on orthopantomogram according to the Levandoski method was 1.12 for the left and 0.91 on the right (Figure 3). It has been previously proposed that if the length ratio is greater than 1.1, further likelihood of coronoid hyperplasia is suspected [14]. An analysis by oral and maxillofacial radiologists validated the initial assessment and did not find additional findings.

**Figure 4:**
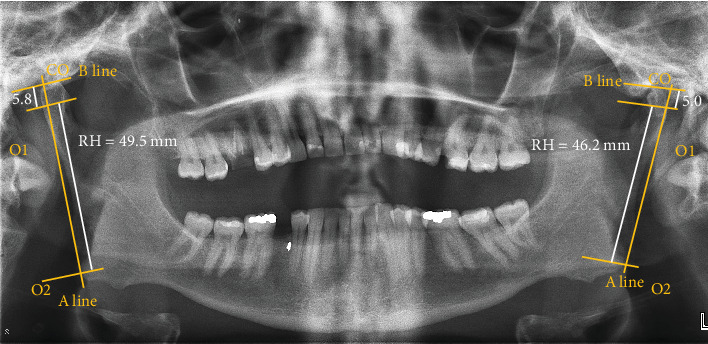
Assessment of facial asymmetry. Linear measurements (CH: condyle height; RH: ramus height) were made by determining specific anatomical points: the highest point of condyle (Co), most lateral point of condyle (O1), most lateral point of ramus (O2), ramus tangent (A line), perpendicular line from A to Co (B line). The asymmetrical index (AI) of condyle and ramus was calculated as follows: AI = [(right − left)/(right + left)] × 100. Following quantification, the condylar AI = 7.4% and ramus AI = 3.44%.

Facial asymmetry was also assessed with the use of radiological imaging (Figure 4). On the radiograph, linear measurements were made by determining specific anatomical points: the highest point of condyle (Co), most lateral point of condyle (O1), most lateral point of ramus (O2), ramus tangent (A line), perpendicular line from A to Co (B line), ramus height (RH), and condyle height (CH). Facial asymmetry was determined based on calculations developed by Habets et al. According to the measurements made, the left condyle and ramus were shorter than the left, with a condylar asymmetry of 7.4% and a ramus symmetry of 3.44% (Figure 4). In reference to the formula developed by Habets et al., an asymmetry index (AI) greater than 3% indicates the presence of asymmetry [15]. Based on the history, clinical examination, and radiographic findings, a diagnosis of left unilateral coronoid hyperplasia and ipsilateral condylar hypoplasia was made. A second clinician validated the diagnosis.

Changes in the size of the mandibular condyle may also result from resorptive bone changes associated with TMJ disorders, such as anterior disc displacement, a diagnosis that requires soft tissue imaging for confirmation.

The patient was in no pain and was unconcerned about his restricted mouth opening since he was comfortably able to perform daily activities. The management of the developmental disorder included educating the patient of his condition and providing instructions to implement jaw mobility while reducing the load on the TMJ. This necessitated the regular and consistent mouth opening exercises, maintaining the opening at rotational or hinge axis.

## 3. Case Report #2

A 27-year-old female of Hispanic origin was referred to the Orofacial Pain Clinic complaining of bilateral jaw pain that commenced after a physical assault (facial trauma) when she was a late teen. She also reports open lock that started happening a few weeks prior her first visit to the clinic.

**Figure 5:**
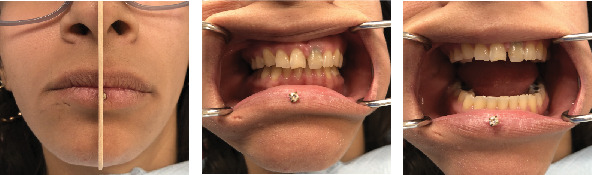
Photographs showing extraoral and intraoral findings.

External clinical examination revealed noticeable facial asymmetry; the chin was deviated (approximately 5 mm) toward the right side related to the craniofacial midline (glabella, subnasale, and pogonion) (Figure 5). No photographic analysis was performed. Masticatory muscles demonstrated no tenderness upon digital palpation, except for the left masseter muscle, which presented with moderate pain. Moreover, no pain was reported in the lateral and dorsal capsules of the right TMJ region, while moderate pain was experience upon palpation of the left side. Intracapsular sounds were not identified, and the mandible appeared to deviate to the right side with lack of right TMJ translation on opening of the mouth. Furthermore, there were a limited range of mandibular movements, a maximum pain-free opening of 25 mm, and a MIO of 40 mm, both maximum assisted and unassisted. The patient was unable to achieve lateral movements of the mandible. The opening path was slightly deflected to the right. Intermittent bilateral facial pain was reported that was aggravated with function (eating and opening mouth). Intraoral normality of alveolar tissues was noted, and bilateral posterior tooth contact was achieved; however, pronounced wear of maxillary and mandibular dentition was observed (Figure 5). The patient did not recall any daytime clenching and was unaware of nighttime grinding.

**Figure 6:**
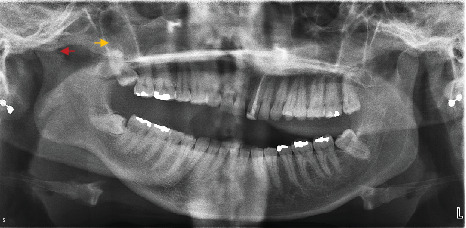
Orthopantomography of the patient. Right coronoid process hyperplastic (yellow arrow). Right condyle hypoplastic (red arrow).

**Figure 7:**
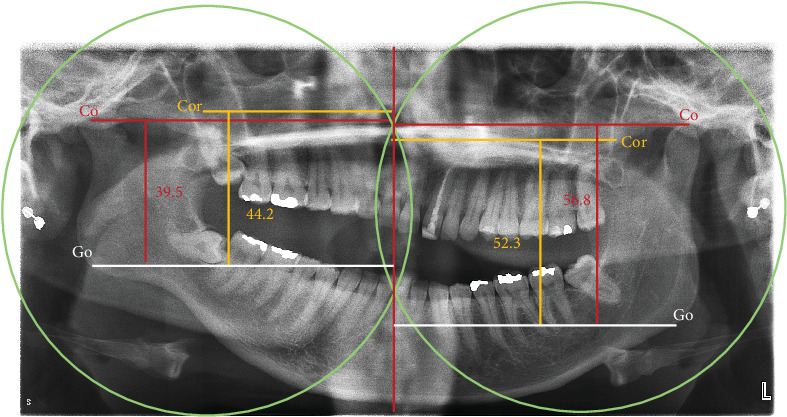
Levandoski method to assess coronoid hyperplasia. Vertical midline was drawn between the two intersections of both arcs (center of arches = maxillary tuberosities/distal height of the second molars). Three horizontal lines were drawn perpendicular to the first line. 1—condyle (Cd) (red, Cd-midline) was determined from the tip of the condyle, 2—coronoid (Cor) (yellow, Cor-midline) was measured from the tip of the coronoid, and 3—gonion (Go, white) across the lower border of the symphysis of the mandible. The Go⁣′–Cor⁣′/Go⁣′–Cd⁣′ length ratio was then calculated. Right = 1.11 and left = 0.92.

**Figure 8:**
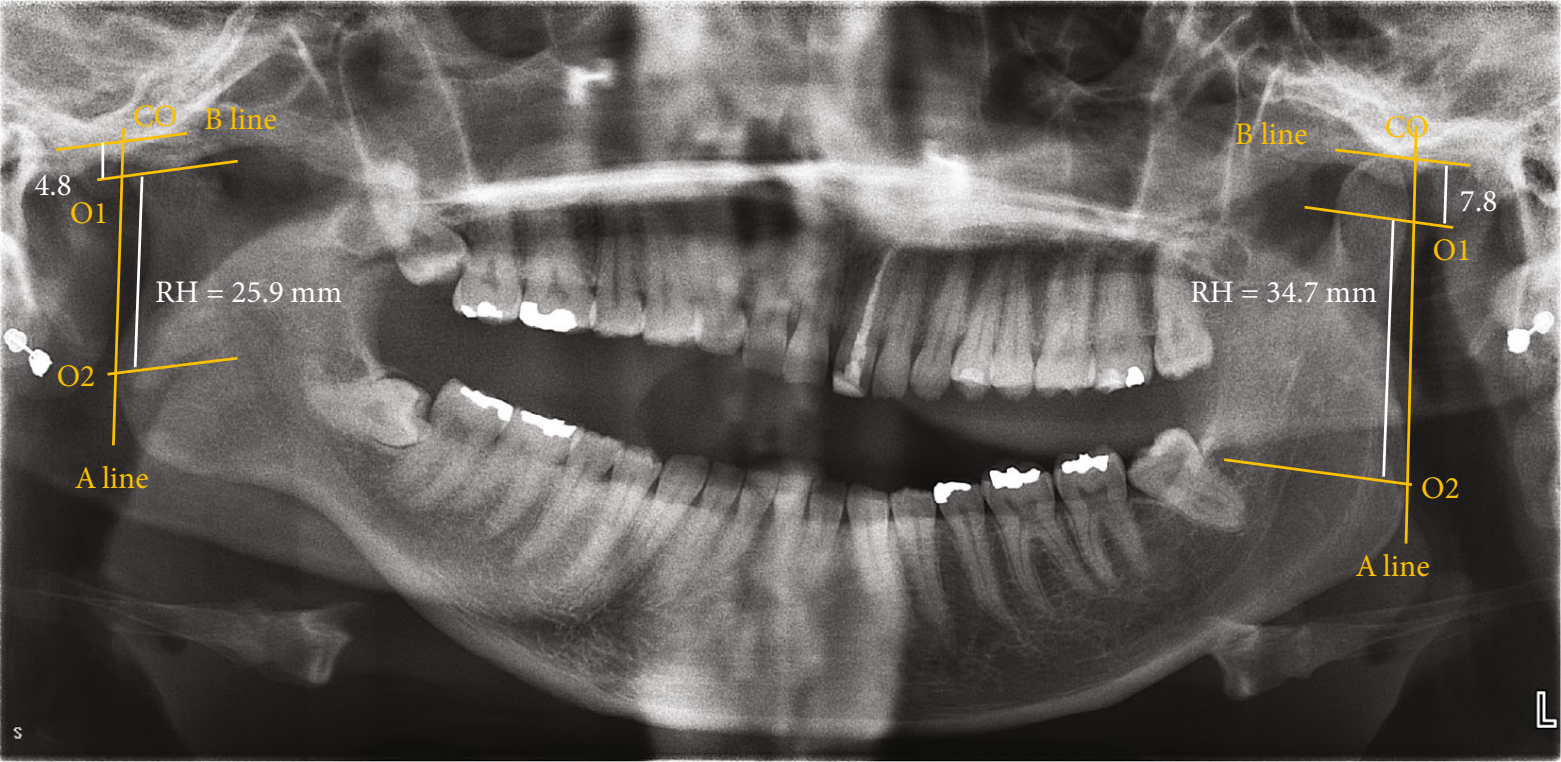
Assessment of facial asymmetry. Linear measurements (CH: condyle height; RH: ramus height) were made by determining specific anatomical points: the highest point of condyle (Co), most lateral point of condyle (O1), most lateral point of ramus (O2), ramus tangent (A line), perpendicular line from A to Co (B line). The asymmetrical index (AI) of condyle and ramus was calculated as follows: AI = [(right − left)/(right + left)] × 100. Following quantification, the condylar AI = 23.8% and ramus AI = 14.5%.

An orthopantomogram was taken revealing a left TMJ with a normal condyle shape and size, which was slightly subluxated beyond the eminence (Figure 6). Right hemimandibular hypoplasia was evident with a deep antegonial notch. The right condyle was hypoplastic and abnormally shaped and involved surface flattening. The condyle remained in the glenoid fossa and lacked translation. Interestingly, the hypertrophy of the right coronoid process can be probably observed (Figure 6). There was no radiographic evidence of any pathology in the coronoid process. The coronoid–condyle length ratio measurement on orthopantomogram was 1.1 for the right and 0.92 on the left (Figure 7). Facial asymmetry was also assessed, and based on the measurements made, the right condyle and ramus were shorter than the left, with a condylar asymmetry of 23.8% and a ramus symmetry of 14.6% (Figure 8). Based on the history, clinical examination, and radiographic findings, a diagnosis of arthralgia, myofascial pain, and right unilateral coronoid hyperplasia and condylar hypoplasia was made. Diagnosis was further validated by a second clinician. Oral and maxillofacial radiologists validated the initial assessment, and no additional findings were confirmed. As with the first reported case, changes in the bony structures of the TMJ may also be the result of bone changes associated with disorders such as anterior disc displacement, and further imaging would be needed to confirm the diagnosis, such as the CBCT.

The patient was educated about the condition; however, she was in outstanding pain that needed attention. Pain management plan was the prescription of a nonsteroidal anti-inflammatory drug (nabumetone, 500 mg bid for 2 weeks) and a skeletal muscle relaxant (cyclobenzaprine, 10 mg for 2 weeks) to relieve muscle spasm. The treatment plan was further supported with home-based therapies and avoidance protocols where the patient is encouraged to restrict the range of motion of the jaw, by cutting food in small pieces and limit opening the mouth wide. Instructions and guidance of thermal therapy to affected muscles and mobility exercises and stretches that would help reduce the load on the TMJ were demonstrated.

At the second visit, the patient felt notable improvement following the medication course. She did not have pain until 3 days prior to her visit when she reported some brief sharp pain. Upon examination, her maximum pain-free opening enhanced to 40 mm (baseline 25 mm) and MIO of 40 mm both maximum assisted and unassisted; however, she was still not able to make lateral mandibular movements. At this visit, no pain was reported on palpation of the right TMJ, which did not translate when opening, and the previously reported moderate pain on the left TMJ was now mild. In addition, no pain or tenderness was described upon digital palpation of masticatory muscles. The patient was reinforced to continue with home-based therapy given at the first visit, as notable progression with stretches and exercises was observed.

On the third visit, the patient reported a complete resolution of pain since her last visit. Following extraoral examination, her maximum pain-free opening remained at 40 mm (baseline 25 mm); however, a MIO of 45 mm both maximum assisted and unassisted was noted. On examining her lateral mandibular movements, though limited, she was able to move her jaw side to side with a maximum right lateral movement of 3 mm and left lateral motion of 4 mm. There was no pain on palpation of TMJ or masticatory muscles. She described her noticing her teeth become shorter. Intraorally, progressive generalized attrition of incisal edges and cusp tips was observed. Maxillary occlusal orthotic appliance was planned, while continuing with the home-based therapy. The patient was very concerned with her facial asymmetry and was keen to have it fixed. Given the complexity of the case, including probable unilateral condylar hypoplasia and significant occlusal wear, the patient was referred to an oral and maxillofacial surgeon for comprehensive surgical evaluation, including assessment for potential bimaxillary orthognathic intervention and further diagnostic workup if indicated.

## 4. Discussion

Unilateral coronoid hyperplasia and condyle hypoplasia, in which trauma during childhood or adolescence is the primary etiology, are rare disorders of mandibular growth. Mandibular condylar hypoplasia is characterized by progressive reduction in the condyle size, which may result in a retrognathic mandible, malocclusion, where premature contact of the affected side and open bite of the contralateral side may be involved, in addition to facial asymmetry. Posttraumatic bone loss primarily involves recruitment of osteoclasts and induction of bone resorption. This osteoclastogenic process is mainly regulated by proinflammatory cytokines, interleukin 6 (IL6), tumor necrosis factor alpha (TNF*α*), and receptor activator nuclear factor kappa-beta ligand (RANKL) [16].

Up to the present day, the etiopathogenesis of coronoid process hyperplasia remains uncertain. Consequently, many hypotheses have been put forward to interpret the progression of this condition. Primarily, bilateral coronoid hyperplasia is clinically manifested in patients with a genetic predisposition. Genetic disorders such as masticatory muscle tendon-aponeurosis hyperplasia and trismus-pseudocamptodactyly syndrome are associated with excessive muscle spasm and coronoid process hyperplasia to the affected site [17, 18]. Enlarged coronoid may also be associated with skeletal muscle hypotonia, such as Moebius syndrome [18, 19], muscular dystrophy [20], Pena–Shokeir syndrome [21], cleidocranial dysplasia [22], Shprintzen–Goldberg syndrome [23], Carey–Fineman–Ziter syndrome [24], Pompe disease [25], and Loeys–Dietz syndrome [26]. It has recently been suggested that Shprintzen–Goldberg syndrome is associated with mutations in the fibrillin-1 (FBN1) gene and SKI gene, while transforming growth factor *β* (*TGFβ*) gene mutation has been linked to Loeys–Dietz syndrome [18]. Cleidocranial dysplasia syndrome, a rare condition that affects dentition and bone, is caused by deletions in the *RUNX2* gene [22]. Finally, mutations in SMAD 2/3 signaling oncogene, was identified in the most disabling hypertrophic ossification disorder, Munchmeyer's disease [27].

Temporomandibular disorders have also been proposed to be linked to coronoid hyperplasia. It is regularly seen simultaneously with untreated TMJ ankylosis. In this situation, to maintain equilibrium of the depressor and elevator musculature of the jaw, some muscles will compensate for others. During mouth opening, the fibrosed tendon of the temporalis muscle induces hyperactivity of the suprahyoid muscle and in return these muscles induce isometric contracture of the temporalis muscle [28, 29]. Eventually, elongation or hyperplasia of the coronoid process develops due to the temporalis muscle hyperactivity, which is equivalent to distraction osteogenesis [13]. In addition, coronoid hyperplasia has been associated with extended TMJ disc derangement [30]. It is a well-established fact that both TMJ disc disorder and unilateral coronoid process hyperplasia cases present with similar clinical features, them being progressive restriction of mouth opening and ipsilateral jaw deviation. Both cases underscore the diagnostic overlap between coronoid hyperplasia and TMJ disorders. Because both conditions can independently or jointly cause limited mouth opening and jaw deviation, a thorough evaluation is critical to distinguishing the primary etiology. Especially prior or without radiographic analysis, some cases are initially misdiagnosed as TMJ disorder depending on patients' clinical manifestations but are later diagnosed as mandibular coronoid process hyperplasia following the discovery of the radiographic findings [31].

Several authors have attributed coronoid hyperplasia to a history of facial trauma, physical injury to the mandible [32, 33] or zygomatic arch [33, 34]. It was proposed that after an individual is subjected to facial trauma, the temporalis muscle exerts tensile forces on the coronoid, promoting distraction osteogenesis resulting in an increase in size and length of the process. Moreover, it has also been postulated that following injury to the temporalis tendon proximal to its insertion results in the development of a hematoma and consequential clot disorganization promoting osteogenesis of the coronoid [35]. Furthermore, it has been reported that an acquired underdeveloped or hypoplastic condyle may be concomitant with hypertrophy of the coronoid process on the affected side [2]. It is important to highlight the functional role of the temporalis muscle. Despite the fact that none of the proposed hypotheses are supported with strong evidence, we can recognize and appreciate that the majority of these hypotheses suggest a pivotal role of the temporalis muscle. This muscle should not be disregarded in the etiopathology of coronoid process hyperplasia, as it is the principal structure attached to the coronoid.

Dental orthopantomogram is the most commonly used imaging procedure to support the diagnosis of bony defect. Levandoski protocol was developed to aid with panoramic analysis. Dimensions of the condyle-gonion and the coronoid-gonion are measured, and the ratio of both these values was quantified. A mean ratio of up to 0.94 was considered within normal limits, while a value ≥ 1.1 qualified as diseased or abnormal. They further attributed that patients with a ration exceeding 1.1 were anticipated to have developed coronoid process hyperplasia [14].

Moreover, CBCT remains the second choice often applied when panoramic topographic radiography has already established the diagnosis [31]. Nonetheless, 3D diagnostic imaging has revolutionized the evaluation of the condyle and coronoid process. Tavassol et al. introduced an effective tool to assess the length of the coronoid process through CT scan [36]. This method provides beneficial insight to the quantitative parameters involved with coronoid hyperplasia. Recently, Mattei et al. compared the morphometric values obtained from orthopantomograms and CT scans and concluded that measurements computed from scanography measurement methods was superior to two-dimensional imaging [37]. Particularly in cases where surgical treatment is pursued, confirming zygomatic–mandibular interference requires axial CT or CBCT imaging. Orthopantomography alone lacks the spatial resolution to assess impingement, and thus, diagnostic certainty may be limited in conservatively managed patients. The 3D reconstruction of the maxillofacial complex through CT or CBCT scan is mandatory to make an accurate presurgical evaluation.

The treatment strategies for mandibular condylar hypoplasia vary depending on the age of the patient. In growing patients, during the presurgical phase, orthopedic management strategies with functional appliances have been reported to be beneficial to correct deformities. After the patient growth stops, surgeons recommend the reconstruction of the mandibular deficiency, sagittal split osteotomy, with or without the alloplastic TMJ replacement. This largely depends on the presence or absence of TMJ-associated symptoms.

The aim of treating coronoid hyperplasia is the rehabilitation and maintenance of adequate long-term mouth opening [38]. Ineffective conservative therapies from misdiagnosed cases can establish distressed unsatisfied patients with a compromised quality of life. Accordingly, early diagnosis is of prime importance. It is necessary to be aware that TMJ disorders and coronoid hyperplasia can be concurrent. Primarily, since coronoid hyperplasia is a mechanical problem due to interference of the coronoid with the zygoma, the current treatment of choice is surgical [39].

Coronoidectomy is a procedure that involves the complete excision of the osteotomized coronoid process that attaches the temporalis muscle. On the other hand, coronoidotomy is a more conservative approach where only the muscle attachment is stripped but the osteotomized coronoid process remains [40]. To prevent muscle reattachment of the coronoid process, modified surgical procedure, gap coronoidotomy, was introduced by Chen et al. [41]. In most cases, the intraoral approach is preferrable. The benefits being getting efficient access, reducing the risk of facial nerve damage and subsequent formation of a visible scar. However, the development of postoperative hematoma and fibrosis can be a possible complication. To minimize this complication, an endoscopically assisted intraoral approach has reported to be favorable [42]. The extraoral approach allows better visibility for the complete stripping of the temporalis muscle to avoid any deviant muscle activity [4].

Continuous and consistent postoperative rehabilitation and physiotherapy is essential to obtain and maintain outcome longevity [43]. Relapse has been reported, where redevelopment of a surgically excised coronoid process has occurred. It has been proposed that the development of hematoma, and later fibrosis due to incorrect reorganization of the hematoma, can lead to an unfavorable prognosis [44]. It is advised to commence with physiotherapy within 1-week postsurgery for a duration of 6 months for optimal results [39]. Long-term rehabilitation is thought to be the most essential variable for long-term success. Another appliance that has been used as a supportive therapy following surgical intervention to improve restricted mouth opening is the TheraBite. This device helps stretch fibrotic tissue in the jaw by repetitive passive motion [40].

## 5. Conclusion

These case reports highlight the link between TMJ disorders and possible unilateral coronoid process hyperplasia, both presenting with progressive mouth opening restriction and ipsilateral jaw deviation. Notably, these conditions may coexist. Accurate diagnosis requires careful clinical evaluation supported by radiographic imaging to distinguish coronoid hyperplasia. Unilateral condylar atrophy with a hyperplastic coronoid is a rare cause of limited mouth opening. Clinical outcomes and patient satisfaction often diverge from objective findings, emphasizing the need for a personalized care. These cases also show a need for further 3-D diagnostic strategy and further research in this area.

## Data Availability

The clinical data supporting this case report are not publicly available due to patient privacy and confidentiality requirements. Deidentified data relevant to the case may be obtained from the corresponding author upon reasonable request. All literature cited in the review is publicly available through the referenced journals and databases.
